# DLK/JNK3 Upregulation Aggravates Hair Cell Senescence in Mice Cochleae via Excessive Autophagy

**DOI:** 10.1111/acel.70099

**Published:** 2025-05-12

**Authors:** Rui Ding, Weiyi Huang, Chenling Shen, Yi Pan, Yiming Zhong, Bing Kong, Yilin Shen, Mingliang Xiang, Bin Ye

**Affiliations:** ^1^ Department of Otolaryngology & Head and Neck Surgery, Ruijin Hospital Shanghai Jiao Tong University School of Medicine Shanghai China; ^2^ Shanghai Key Laboratory of Translational Medicine on Ear and Nose Diseases Shanghai China; ^3^ Ear Institute Shanghai Jiao Tong University School of Medicine Shanghai China; ^4^ Department of Audiology & Speech‐Language Pathology, College of Health Science and Technology Shanghai Jiao Tong University School of Medicine Shanghai China

**Keywords:** age‐related hearing loss, autophagy, hair cell senescence, mitogen‐activated protein kinase, ubiquitin‐proteasome system

## Abstract

Cell death mediated by the abnormal activation of autophagy has been observed in many neurodegenerative diseases. Dual leucine zipper kinase (DLK), a member of the mitogen‐activated protein kinase cascade, plays a key role in regulating cellular autophagy and the progression of neurodegenerative diseases. However, its role in age‐related hearing loss has not been reported. In this study, we found that DLK, phosphorylated c‐Jun N‐terminal kinase (p‐JNK), and JNK3 expression increased in the cochleae of C57BL/6J mice during aging. The DLK/JNK pathway and autophagy are excessively activated in the House Ear Institute‐Organ of Corti 1 (HEI‐OC1) senescent hair cell line. After DLK was upregulated in HEI‐OC1 cells, autophagy was activated, and cell aging was initiated. Inhibiting the DLK/JNK pathway in senescent HEI‐OC1 cells can reduce autophagy activation and senescence, and inhibiting autophagy activation can also alleviate senescence. The inhibition of DLK or JNK3 in vivo significantly reduced age‐related cochlear structural damage and hearing loss in C57BL/6J mice. The results of the present study showed that DLK/JNK3 may play a key role in cochlear hair cell senescence and age‐related hearing loss through the abnormal activation of autophagy within cochlear hair cells, suggesting that DLK or JNK3 may be potential targets for alleviating age‐related hearing loss.

## Graphical abstract

In normal physiological processes, autophagy acts as a protective factor against aging stress. With the stress increase, the DLK/JNK3 pathway is activated and autophagy is continuously upregulated. In our study, excessive autophagy leads to hair cell senescence, which reveals that excessive autophagy can play a harmful role in senescence under certain conditions.

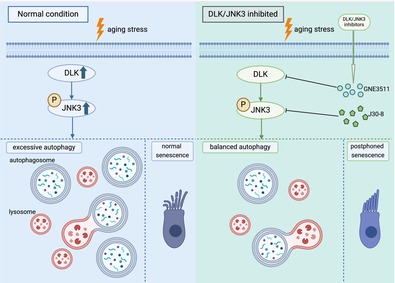

**Abbreviations**
3‐MA: 3‐methyladenine
ABR: auditory brainstem response
ARHL: age‐related hearing loss
ATG5: autophagy protein 5
DLK: dual leucine zipper kinase
HEI‐OC1: House Ear Institute‐Organ of Corti 1
LC3B: microtubule‐associated proteins 1A/1B light chain 3B
MAPK: mitogen‐activated protein kinase
PBS: phosphate‐buffered saline
p‐JNK: phosphorylated c‐Jun N‐terminal kinase
ULK1: Unc‐51‐like autophagy‐activating kinase 1
UPS: ubiquitin‐proteasome system

## 1 Introduction

Aging is a global tendency of social development. Age‐related hearing loss (ARHL), a disease that seriously affects the quality of life of the elderly, has recently been brought into focus. However, its pathogenesis remains unclear.

The mitogen‐activated protein kinase (MAPK) pathway is a common signal transduction pathway in various physiological and pathological processes and mainly comprises three signal levels: MAPK kinase kinase, MAPK kinase, and MAPK (Burotto et al. 2014). MAPKs play multiple roles in hearing development (Kantarci et al. 2020), maintenance (Yousaf et al. 2015), and damage (Tang, Hu, et al. 2021). As third effectors, MAPKs, mainly extracellular signal‐regulated kinase (ERK)1/2, c‐Jun N‐terminal kinase (JNK)1/2/3, and p38, receive various upstream signals and transduce downstream transcription factors to initiate apoptosis, proliferation, and other processes (Burotto et al. 2014). Currently, in audiology, research on MAPKs mainly focuses on ototoxic deafness and noise‐induced hearing loss. Several studies have shown that conditional ERK2 knockout in cochlear hair cells does not affect basic hearing in mice but makes hair cells more vulnerable to noise, indicating that ERK2 might protect hair cells from noise exposure (Kurioka et al. 2015). Kanamycin causes ototoxicity by inducing high phosphorylated p38 and phosphorylated JNK (p‐JNK) expression in hair cells. α‐Lipoic acid attenuates kanamycin ototoxicity by inhibiting p38 and JNK phosphorylation (Wang et al. 2012). The JNK pathway is activated in cisplatin‐induced hair cell injury, and phosphatase and tensin homolog‐induced kinase 1 inhibits this pathway, thus playing a protective role (Yang et al. 2018). However, few reports on the role of MAPK in ARHL are available.

In the present study, we focused on the mechanism of the MAPK pathway in ARHL. Our previous studies identified the different characteristics of different JNK subtypes during the aging process of cochlear hair cells in C57BL/6J mice, suggesting that JNK3 contributes the most to hair cell senescence (Ding et al. 2023). JNK3 expression is highly selective and especially high in neurons compared with other cell types (Kumar et al. 2015), indicating that it may play an important role in neuronal development, maturation, and related diseases. Also, JNK3 is an important member of the MAPK pathway. By analyzing single‐cell sequencing results of cochlear hair cells from C57BL/6J mice at different months from Chai et al. (Sun et al. 2023), we found that the MAPK pathway was significantly upregulated in ARHL and enriched the most differentially expressed genes. However, the upstream regulators of JNK and whether their regulation alleviates ARHL via the JNK pathway remain to be established. Therefore, this study aims to explore the upstream mechanism regulating hair cell senescence.

In recent years, many studies have shown that dual leucine zipper kinase (DLK, also known as MAP3K12) is an important biomarker and a potential target in neurodegenerative diseases (Le Pichon et al. 2017; Siu et al. 2018; Welsbie et al. 2017). DLK expression is significantly upregulated in classic neurodegenerative diseases such as Alzheimer's (Hayne and DiAntonio 2022) and Parkinson's disease (Tu et al. 2021), amyotrophic lateral sclerosis (Le Pichon et al. 2017), glaucoma‐induced optic ganglion degeneration (Welsbie et al. 2017), and glutamate‐induced neuronal excitability poisoning (Fahrenthold et al. 2018). Pharmacological inhibition and gene knockout of DLK can reduce the apoptosis of demyelinated retinal ganglion cells in mice, suggesting that demyelinating diseases can be prevented by inhibiting DLK signaling in neurons (Duncan et al. 2023). However, no studies on the cellular mechanisms of DLK in cochlear hair cell senescence in ARHL are available.

## 2 Materials and Methods

### 2.1 Experimental Animals

C57BL/6J mice were purchased from SPF (Suzhou, China) Experimental Animal Technology Co. Ltd. and raised in specific pathogen‐free animal rooms. Twenty 6‐month‐old C57BL/6J mice were intragastrically administered 30 mg/kg GNE3511 every other day, and ten 6‐month‐old C57BL/6J mice were intraperitoneally administered 30 mg/kg J30‐8 every other day. All animal experiments were approved by the Animal Ethics Committee of Ruijin Hospital, Shanghai Jiao Tong University School of Medicine.

### 2.2 Auditory Brainstem Response

All mice were examined using the ABR in a soundproof room to evaluate their auditory function. Ketamine (80 mg/kg) was used to induce general anesthesia. The needle electrodes were inserted into the middle of the skull (+ electrode, recording electrode), mastoid (− electrode, reference electrode), and hind leg (grounding electrode). Tone‐burst stimulation was provided using a loudspeaker at 4, 8, 11, 16, and 22 kHz and repeated stimulation with 5‐dB decrements starting from 90 dB. A Tucker‐Davis Technologies (Alachua, FL, USA) system was used for recording.

### 2.3 Obtaining the Cochlea

After the ABR examination, the mice were sacrificed, and the bilateral cochlea was removed using anatomical forceps. Excess tissues were removed under a stereoscopic microscope, and the cochleae were immersed in 4% paraformaldehyde for 24 h. After fixation, the cochleae were transferred to 10% EDTA for decalcification, and the solution was changed every other day until the bony portion of the vestibule was easily pierced with a needle.

### 2.4 Cochlear Whole Mount and Immunofluorescence

The cochlear whole mount was divided into basal, middle, and apical turns. The following antibodies were used for immunofluorescence analysis: Prestin (Santa Cruz Biotechnology, sc‐293212; 1:500), vglut3 (Synaptic Systems, 135203; 1:500), p‐JNK (Santa Cruz Biotechnology, sc‐6254; 1:300), donkey anti‐rabbit 568 (Thermo Fisher Scientific, A10042; 1:500), and goat anti‐mouse 488 (Invitrogen, A11001; 1:500). To stain cell nuclei, the samples were counterstained with Hoechst 33342 (Thermo Fisher Scientific, H3570; 1:5000). Images were acquired using a NiE‐A1 Plus confocal microscope (Nikon, Tokyo, Japan) at 100× and 600× magnifications.

### 2.5 Paraffin Sectioning and Immunohistochemistry Staining

Each decalcified cochlea was dehydrated in graded ethanol, infiltrated in xylene, embedded in paraffin at 25°C, and cut into 4‐μm slices. The paraffin sections were deparaffinized in xylene and ethanol, immersed in antigen retrieval buffer, and baked. After washing thrice with phosphate‐buffered saline (PBS), the slices were placed in a 3% hydrogen dioxide solution and incubated for 25 min at 25°C in the dark. Subsequently, cells were blocked with 3% bovine serum albumin for 30 min at 25°C. The blocking buffer was removed, and the slices were incubated with the following primary antibodies after PBS dilution: DLK (GeneTex, GTX124127; 1:100), p‐JNK (Cell Signaling Technology, 4668S; 1:100), ULK1 (Proteintech, 29005‐1‐AP; 1:100), and ATG5 (Abmart, T55766; 1:100) overnight at 4°C. After removing the primary antibodies, horseradish peroxidase‐linked secondary antibodies (Servicebio, GB23301 and GB23303; 1:200) from the same species as the primary antibodies were added, and the slices were incubated for 50 min at 25°C. Finally, a 3,3′‐diaminobenzidine solution was added, and the reaction was stopped using a wash buffer when appropriate. Images were scanned and viewed using the Caseviewer software (3DHISTECH, Budapest, Hungary) at 55× magnification.

### 2.6 Cell Culture

HEI‐OC1 cells were cultured in high‐glucose DMEM containing 10% fetal bovine serum and 1% penicillin in a humid atmosphere of 5% CO_2_ and 95% air at 37°C. To induce cellular senescence, hair cells were treated with d‐galactose (Sigma, G5388) solution. We prepared a 400 mM stock solution by dissolving the powder in complete culture medium with vortexing.

### 2.7 Cell Protein Extraction and Western Blot

Total protein from each group was extracted using RIPA lysis buffer (Epizyme Biotech, PC101) supplemented with protease and phosphatase inhibitors (MedChemExpress, HY‐K0010, HY‐K0021, HY‐K0022, HY‐K0023; 1:100). The total proteins were lysed on ice for 30 min and centrifuged at 4°C at 13,200*g*. The protein concentration of the supernatant was determined using BCA (Epizyme Biotech, ZJ102), and the proteins were transferred to a PVDF membrane following SDS‐polyacrylamide gel electrophoresis. The membrane was blocked with TBST containing 5% bovine serum albumin for 1 h and washed thrice. The following primary antibodies were used: DLK (GeneTex, GTX124127; 1:1500), JNK3 (Cell Signaling Technology, 2305S; 1:1000), p‐JNK (Cell Signaling Technology, 4668S; 1:1500), LC3B (Abmart, T55992; 1:1500), ATG5 (Abmart, T55766; 1:1500), p62 (Abmart, T55546; 1:1500), ULK1 (Proteintech, 29005‐1‐AP; 1:2000), Beclin‐1 (Proteintech, 11306‐1‐AP; 1:2000), p21 (Proteintech, 10355‐1‐AP; 1:2000), p53 (Proteintech, 60283‐2‐Ig; 1:2000), p16 (Affinity, AF5484; 1:1500), Lamin B1 (Proteintech, 66095‐1‐Ig; 1:6000), γ‐H2A.X (Servicebio, GB111841‐100; 1:800), Ubiquitin (Servicebio, GB115700‐100; 1:800), GAPDH (Servicebio, GB11002; 1:3000), and β‐actin (Proteintech, 81115‐1‐RR; 1:2000). The membrane was incubated at 4°C overnight. After washing with TBST, a secondary antibody (Cell Signaling Technology, 7074S; 1:5000; Servicebio, GB23301; 1:5000) was added, incubated at 25°C for 1 h, and developed using ECL photoluminescence (Epizymebiotech, SQ203).

### 2.8 Cellular Immunofluorescence

A cell glass slide with a diameter of 20 mm was placed in a 6‐well plate, onto which HEI‐OC1 cells were seeded (5 × 10^4^ cells, 800 μL per well) and incubated overnight under the aforementioned cell culture conditions. After the cells adhered to the slide, 2 mL/well of medium was added. Subsequently, the cells were treated with 200 mM d‐galactose for 48 h, after which the culture medium was washed thrice with PBS. Afterward, the cells were fixed with a paraformaldehyde solution at 25°C for 10 min and then washed thrice with PBS. Next, PBS containing 0.3% Triton‐X and 10% donkey serum was used for blocking and cell membrane disruption for 30 min. After washing thrice with PBS, the primary antibodies were added and incubated overnight at 4°C. The following primary antibodies were used: DLK (GeneTex, GTX124127; 1:100), p‐JNK (Cell Signaling Technology, 4668S; 1:100), p21 (Proteintech, 10355‐1‐AP; 1:200), p16 (Affinity, AF5484; 1:150), p53 (Proteintech, 60283‐2‐Ig; 1:200), ULK1 (Proteintech, 29005‐1‐AP; 1:200), p62 (Abmart, T55546; 1:150), Beclin‐1 (Proteintech, 11306‐1‐AP; 1:200), and LC3B (Abmart, T55992; 1:150). After removing the primary antibodies and washing thrice with PBS, the secondary antibody (Yeasen, 34112ES60, 34206ES60, 34212ES60; 1:300) was added and incubated in the dark at 25°C for 1 h. Thereafter, cytoskeletons were stained with phalloidin (Yeasen, 40734ES75; 1:300) at 25°C for 30 min. Finally, the cells were incubated with 0.5 μg/mL DAPI for 10 min and then washed thrice with PBS. Images were acquired using a microscope (Nikon) at 200× magnification.

### 2.9 β‐Galactosidase Staining

A Cell Senescence β‐galactosidase Staining Kit (Yeasen, 40754ES60) was used for staining. The culture medium was removed, and cells were washed with PBS. The fixing solution (1 mL/well) was added for 15 min at 25°C and then removed Next, a pre‐heated working solution, comprising 10 μL reagent A, 10 μL reagent B, 930 μL reagent C, and 50 μL Xgal solution, was added into each well. The 6‐well plate was sealed with sealing film and incubated at 37°C overnight. Subsequently, the working solution was removed, and each well was washed thrice. Images were acquired using a microscope (Nikon) at 100×, 200×, and 400× magnifications.

### 2.10 Mitochondrial Membrane Potential JC‐1 Staining

A JC‐1 Mitochondrial Membrane Potential Assay Kit (Yeasen, 40706ES60) was used for staining. Briefly, JC‐1 was diluted by adding 50 μL JC‐1 (200×) to 8 mL double distilled water (ddH_2_O) and vortexed. Next, 2 mL JC‐1 buffer (5×) was added and mixed with the working buffer. Thereafter, the culture medium was replaced by 1 mL of culture medium and 1 mL of working buffer, and the 6‐well plate was incubated for 20 min at 37°C. After incubation, the supernatant was removed, and each well was washed with JC‐1 buffer (1×). Afterward, the buffer was removed, and 2 mL of culture medium was added. Images were acquired using a microscope (Nikon) at 200× and 400× magnifications.

### 2.11 Lentivirus Transfection

PGMLV‐CMV‐Mouse_Map3k12‐3×Flag‐EF1‐ZsGreen1‐T2A‐Puro lentivirus (custom made) and GFP‐mRFP‐LC3‐Puro lentivirus plasmids (GM‐0220IV211) were purchased from Genomeditech (Shanghai) Co. Ltd. HEI‐OC1 cells were seeded in 12‐well plates at a density of 1 × 10^5^ cells/well. After the cells adhered, the virus was diluted in 500 μL culture medium (multiplicity of infection = 1:100). After infection for 16 h, the culture medium containing the lentivirus was completely replaced with 1 mL of complete culture medium, and the cells were cultured for 48 h. Thereafter, the cells were selected using 2 μg/mL puromycin for 3 days. Subsequently, the cells were cultured with complete culture medium with 1 μg/mL puromycin. Western blotting was performed to verify the effects of gene overexpression.

### 2.12 Statistical Analysis

GraphPad Prism 8.3 (GraphPad Software, La Jolla, CA, USA) was used for statistical analysis. The hearing data for each group were analyzed using one‐way ANOVA. Image‐Pro Plus (Media Cybernetics Inc., Rockville, MD, USA) was used to calculate the mean density of immunohistochemical staining, and one‐way ANOVA was performed on the data for each group. The average gray values of immunofluorescence and western blotting were calculated using ImageJ (National Institutes of Health, Bethesda, MD, USA), and the data for each group were analyzed by one‐way ANOVA. An unpaired *t*‐test was applied to analyze the differences between two groups. The data are presented as mean ± standard deviation (SD). *p* < 0.05 was considered statistically significant.

## 3 Results

### 3.1 Hearing Threshold Increases Gradually, and Hair Cells Are Lost in Aging C57BL/6J Mice

**FIGURE 1:**
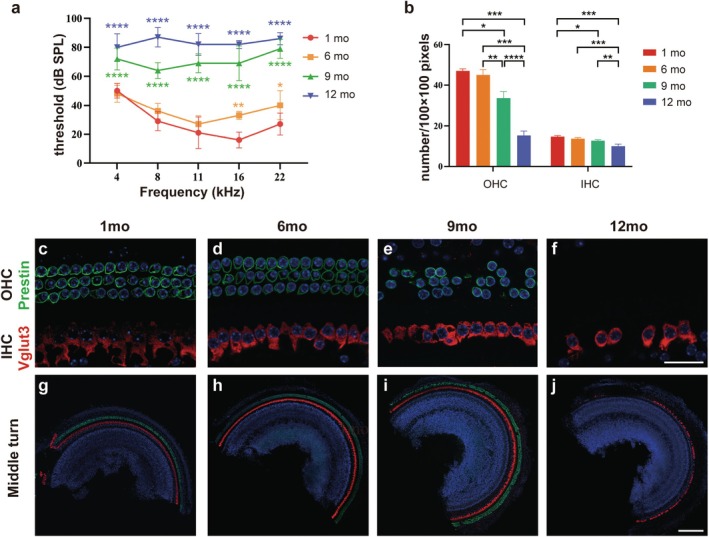
Hearing threshold increased gradually, and hair cells were lost in the aging process of C57BL/6J mice. (a) Compared with that for mice aged 1 month, the ABR threshold at 4, 8, 11, 16, and 22 kHz in C57BL/6J mice increased significantly from 9 months of age. (b) Number of OHCs and IHCs of C57BL/6J mice in (c–j) was counted. (c–j) Arrangement of hair cells in the cochlea of 1‐, 6‐, 9‐, and 12‐month‐old C57BL/6J mice. OHC and IHC loss were significant at 9 and 12 months of age, respectively. Data are shown as mean ± SD, *n* = 5 (a), *n* = 3 (b). *p < 0.05, **p < 0.01, ***p < 0.001, ****p < 0.0001. ABR, auditory brainstem response; IHCs, inner hair cells; mo, month; OHCs, outer hair cells. Scale bar, 20 μm (c–f), 200 μm (g–j).

The auditory brainstem response (ABR) thresholds of 1‐, 6‐, 9‐, and 12‐month‐old C57BL/6J wild‐type male mice were measured. According to the statistics of their hearing thresholds at 4, 8, 11, 16, and 22 kHz (Figure 1a,b), after 6 months of age, the hearing of C57 mice began to deteriorate, worsening with age. From 6 to 9 months old, the threshold increased the most at all tested frequencies.

C57BL/6J mice are the most commonly used animal model for ARHL studies. Cochlear whole mounts of 1‐, 6‐, 9‐, and 12‐month‐old C57BL/6J mice were stained. Outer and inner hair cells were labeled with prestin and Vglut3, respectively. The middle turn is presented as a representative image (Figure 1c–j). The results showed that outer hair cells were lost at 9 months of age and almost disappeared at 12 months, whereas inner hair cells were lost at 12 months of age (Figure 1b). Outer and inner hair cells were counted in an area of 100 × 100 pixels at approximately 25%, 50%, and 75% of the cochlear whole mount in each group. The numbers of outer and inner hair cells in 1‐ and 6‐month‐old mice did not differ significantly.

### 3.2 DLK/JNK3 Pathway and Autophagy in C57BL/6J Mice Cochlea Are Significantly Upregulated During Aging

**FIGURE 2:**
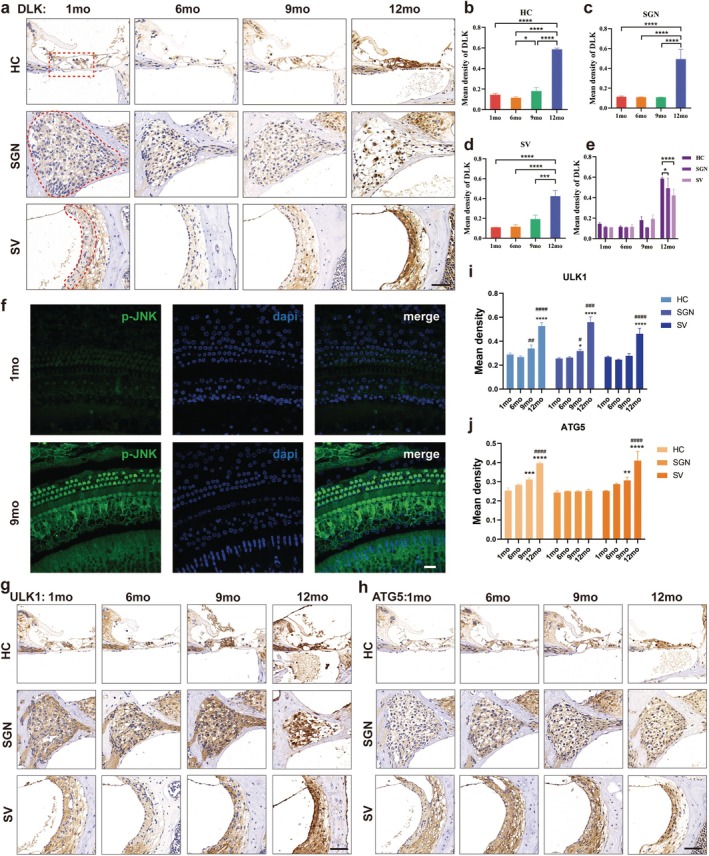
DLK/JNK pathway and autophagy in C57BL/6J mouse cochlea were significantly upregulated with aging. (a) Immunohistochemical staining showed that the expression of DLK in cochlear hair cells, spiral ganglion, and stria vascularis increased gradually with aging. (b–e) Expression intensity of DLK in (a) with aging was counted. (f) p‐JNK immunofluorescence staining of cochlear whole‐mount at 1 and 9 months of age in aging mice. (g, h) Immunohistochemical staining showed that the expression of ULK1 (g) and ATG5 (h) in cochlear hair cells, spiral ganglion, and stria vascularis increased from 1 to 9 months old. (i, j) Statistics of the expression intensity of ULK1 (i) and ATG5 (j) in different parts with aging. * indicates comparison with 1‐month‐old mice, and # indicates comparison with 6‐month‐old mice. Data are expressed as mean ± SD, *n* = 3, *p < 0.05, **p < 0.01, ***p < 0.001, ****p < 0.0001. HC, hair cells; mo, month; SGN, spiral ganglion; SV, stria vascularis. Scale bar, 20 μm (f), 50 μm (a, g, h).

The cochleae of 1‐, 6‐, 9‐, and 12‐month‐old C57BL/6J mice were used for DLK immunohistochemical staining (Figure 2a). DLK was expressed in the hair cells, spiral ganglion, and stria vascularis, and its staining intensity was significantly upregulated after aging. The expression of DLK in hair cells at 9 months of age was significantly higher than that at 6 months, whereas its expression at 12 months of age was significantly higher than that at 1, 6, and 9 months (Figure 2b). In the spiral ganglion and stria vascularis, DLK expression remained stable at 1, 6, and 9 months of age and significantly increased at 12 months (Figure 2c,d). In addition, the expression of DLK in hair cells was significantly higher than that in the spiral ganglion and stria vascularis at 12 months of age, with no significant differences at 1, 6, and 9 months of age (Figure 2e). The results demonstrated that in the aging cochlea, DLK was most significantly expressed in hair cells, and during aging, its upregulation occurred the earliest in hair cells.

p‐JNK is the active form of the JNK protein. Immunofluorescence staining of cochlear whole mounts showed that the expression of p‐JNK in 9‐month‐old mice was significantly higher than that in 1‐month‐old mice, especially in hair cells (Figure 2f), indicating that hair cells expressed more p‐JNK in the cochlea of aging mice. Unfortunately, determining which p‐JNK subtypes are most significantly upregulated in senescent cochlear hair cells is not feasible because of the lack of phosphorylated antibodies against JNK isoforms. However, in our previous study, we found that the most significantly upregulated JNK subtype in the cochlear senescent hair cells of C57BL/6J mice was JNK3, suggesting that the DLK/JNK3 signaling pathway is likely to be significantly upregulated and plays an important role in the aging process of the mouse cochlea.

The expression of the autophagy proteins Unc‐51‐like autophagy‐activating kinase 1 (ULK1, Figure 2g) and autophagy protein 5 (ATG5, Figure 2h) in the mouse cochlea was observed by immunohistochemical staining. The expression of ULK1 in hair cells and the spiral ganglion at 9 and 12 months of age was significantly higher than that at 6 months of age (Figure 2i). ATG5 expression increased significantly in hair cells and the stria vascularis from 1 to 9 months and 6 to 12 months of age, although no significant change in the spiral ganglion during aging was observed (Figure 2j). Overall, autophagy in hair cells is activated by aging in mice.

### 3.3 DLK/JNK3 Pathway Is Upregulated in Senescent Hair Cell Line Model

**FIGURE 3:**
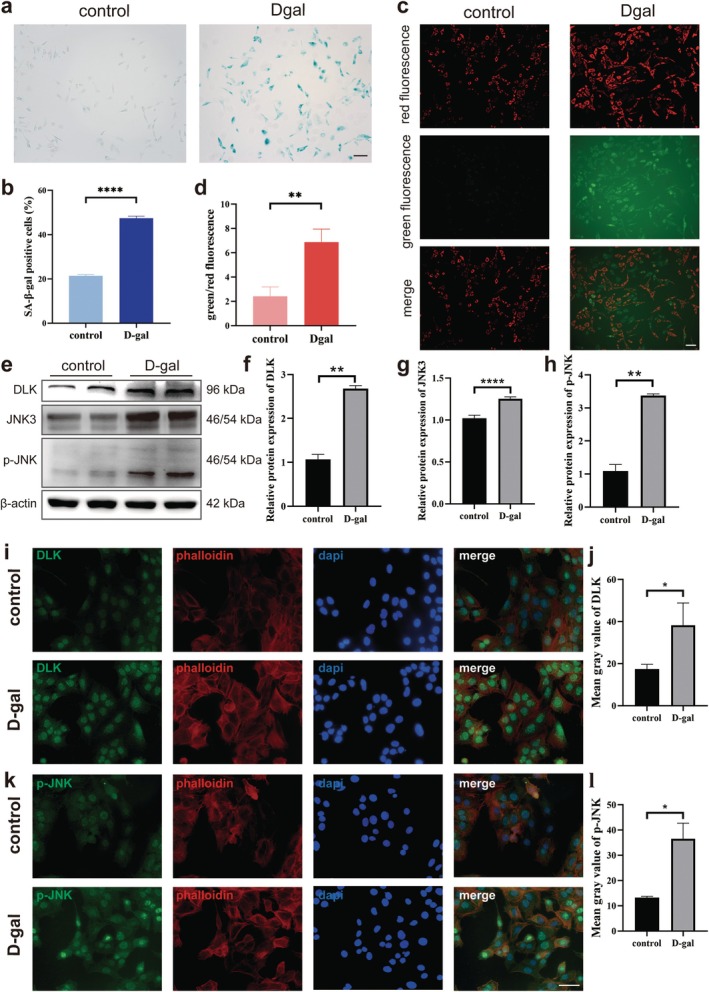
DLK/JNK3 pathway was upregulated in the senescent hair cell line model. (a, c) After HEI‐OC1 cell lines were treated with d‐galactose, the proportion of galactosidase‐positive cells increased. (b, d) After HEI‐OC1 cells were treated with d‐galactose, the mitochondrial membrane potential and ratio of green to red fluorescence intensity increased. (e–h) Western blotting showing that the expression of DLK, JNK3, and p‐JNK increased significantly after d‐galactose treatment. (i–l) Immunofluorescence staining showing that the expression of DLK and p‐JNK increased after d‐galactose treatment. Data are expressed as mean ± SD, *n* = 3. *p < 0.05, **p < 0.01, ****p < 0.0001; mo, month. Scale bar, 20 μm.

After treatment with d‐galactose, a classic senescence inducer, the senescence‐related β‐galactosidase in HEI‐OC1 cells increased significantly and showed sky‐blue deposition in the cytoplasm (Figure 3a). By counting the percentage of β‐galactosidase‐positive cells under three random fields, the percentage of β‐galactosidase‐positive cells in the d‐galactose treatment group was observed to be higher than that in the control group (Figure 3b).

Senescent cells also displayed decreased mitochondrial membrane potential, a marker of increased cell senescence (Figure 3c). The ratio of green to red fluorescence intensity was found to increase significantly in the d‐galactose‐treated group (Figure 3d). Western blotting results showed that, compared with the control group, the expression of DLK, JNK3, and p‐JNK increased significantly after d‐galactose treatment (Figure 3e–h). Immunofluorescence staining showed that the expression of DLK (Figure 3i,j) and p‐JNK (Figure 3k,l) significantly increased in aging HEI‐OC1 cells treated with d‐galactose.

### 3.4 Autophagy Is Excessively Activated in Senescent Hair Cell Line Model

**FIGURE 4:**
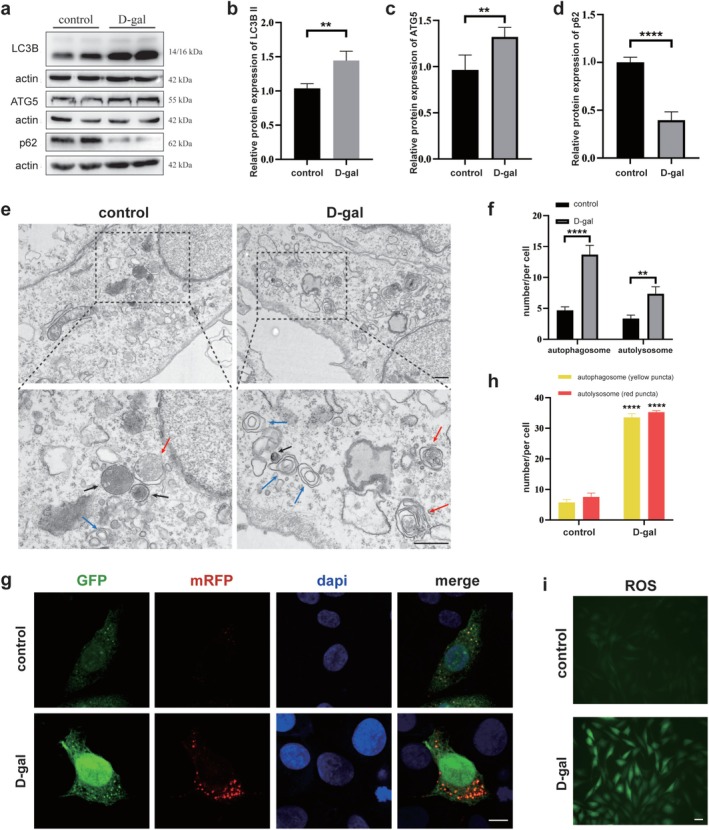
Autophagy was significantly activated in the senescent hair cell line model. (a–d) Western blotting showing that the level of autophagy increased after HEI‐OC1 cells were treated with d‐galactose. (e) Lysosomes (black arrow), autophagosomes (blue arrow), and autolysosomes (red arrow) were detected by transmission electron microscopy. (f) Number of autophagosomes and autolysosomes in a single cell in (e) increased significantly after d‐galactose treatment. (g) Autophagosomes (yellow spots) and autolysosomes (red spots) in each group were observed by confocal microscopy after mRFP‐GFP‐LC3 lentivirus transfection into HEI‐OC1 cells and treatment with d‐galactose. (h) Number of yellow and red spots in a single cell in each group in (g). (i) ROS level of the control and the d‐galactose treatment groups were detected. Data are presented as mean ± SD, *n* = 3. **p < 0.01, ****p < 0.0001. Scale, 5 μm (e, g), 20 μm (i).

After d‐galactose treatment, the expression of autophagy‐related proteins in HEI‐OC1 cells was determined by western blotting (Figure 4a). The results showed that microtubule‐associated proteins 1A/1B light chain 3B (LC3B), ATG5, and SQSTM1/p62 (hereafter referred to as p62) expression levels were significantly higher after d‐galactose treatment than those in the control group (Figure 4b–d). The numbers of lysosomes (black arrows), autophagosomes (blue arrows), and autolysosomes (red arrows) in HEI‐OC1 cells were determined by electron microscopy (Figure 4e). Autophagosomes and autolysosomes in a single HEI‐OC1 cell in the d‐galactose‐treated group were much higher than those in the control group (Figure 4f). HEI‐OC1 cells were transfected with mRFP‐GFP‐LC3 lentivirus and then treated with d‐galactose. Autophagosomes (yellow spots) and autolysosomes (red spots) in a single cell were much higher than those in the control group (Figure 4g,h). Besides, the d‐galactose‐treated group showed markedly elevated reactive oxygen species (ROS) levels compared to the control group (Figure 4i). The results showed that autophagy was excessively activated in the d‐galactose‐induced senescent hair cell line.

### 3.5 Autophagy Is Excessively Activated, and Hair Cell Senescence Is Aggravated due to DLK Overexpression

**FIGURE 5:**
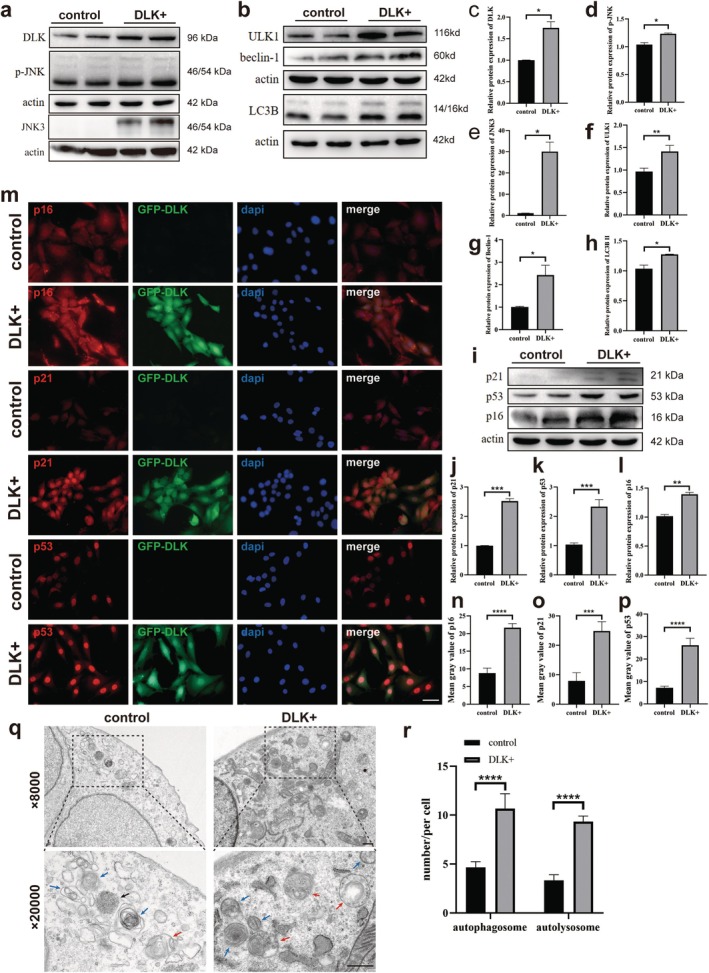
Autophagy was excessively activated, and hair cell senescence was aggravated by DLK overexpression. (a, c–e) Western blot showing that DLK, p‐JNK, and JNK3 were upregulated after DLK overexpression. (b, f–h) Western blot showing that the expression of ULK1, Beclin‐1, and LC3B increased after DLK overexpression. (i–l) Western blot showing that the expression of p21, p16, and p53 increased after DLK overexpression. (m–p) Immunofluorescence showing that the expression of p21, p16, and p53 increased in HEI‐OC1 cells following DLK overexpression. (q) Transmission electron microscopy image showing that autophagosomes (blue arrows) and autolysosomes (red arrows) increased in HEI‐OC1 cells after DLK overexpression. (r) Number of autophagosomes and autolysosomes in a single cell in (p). Data are presented as mean ± SD, *n* = 3. *p < 0.05, **p < 0.01, ***p < 0.001, ****p < 0.0001. Scale, 5 μm.

A GFP‐labeled DLK‐overexpressing HEI‐OC1 cell line was constructed. Compared with the control group, DLK, p‐JNK, and JNK3 expression was significantly upregulated (Figure 5a,c–e). Simultaneously, the expression of the autophagy‐related proteins ULK1, Beclin‐1, and LC3B increased significantly in DLK‐overexpressed HEI‐OC1 cells (Figure 5b,f–h), and the senescence marker proteins p21, p53, and p16 also increased significantly (Figure 5i–l).

Immunofluorescence staining showed that the expression of ULK1, p62, Beclin‐1, and LC3B (Figure S1) and p16, p21, and p53 (Figure 5m–p) increased significantly in DLK‐overexpressing HEI‐OC1 cells. Transmission electron microscopy showed that autophagosomes and autolysosomes were more abundant in DLK‐overexpressing HEI‐OC1 cells than in the control group (Figure 5q,r). These results suggest that autophagy is excessively activated and that HEI‐OC1 senescence is aggravated following DLK overexpression.

### 3.6 DLK/JNK3 Pathway Regulation Affects Hair Cell Senescence

**FIGURE 6:**
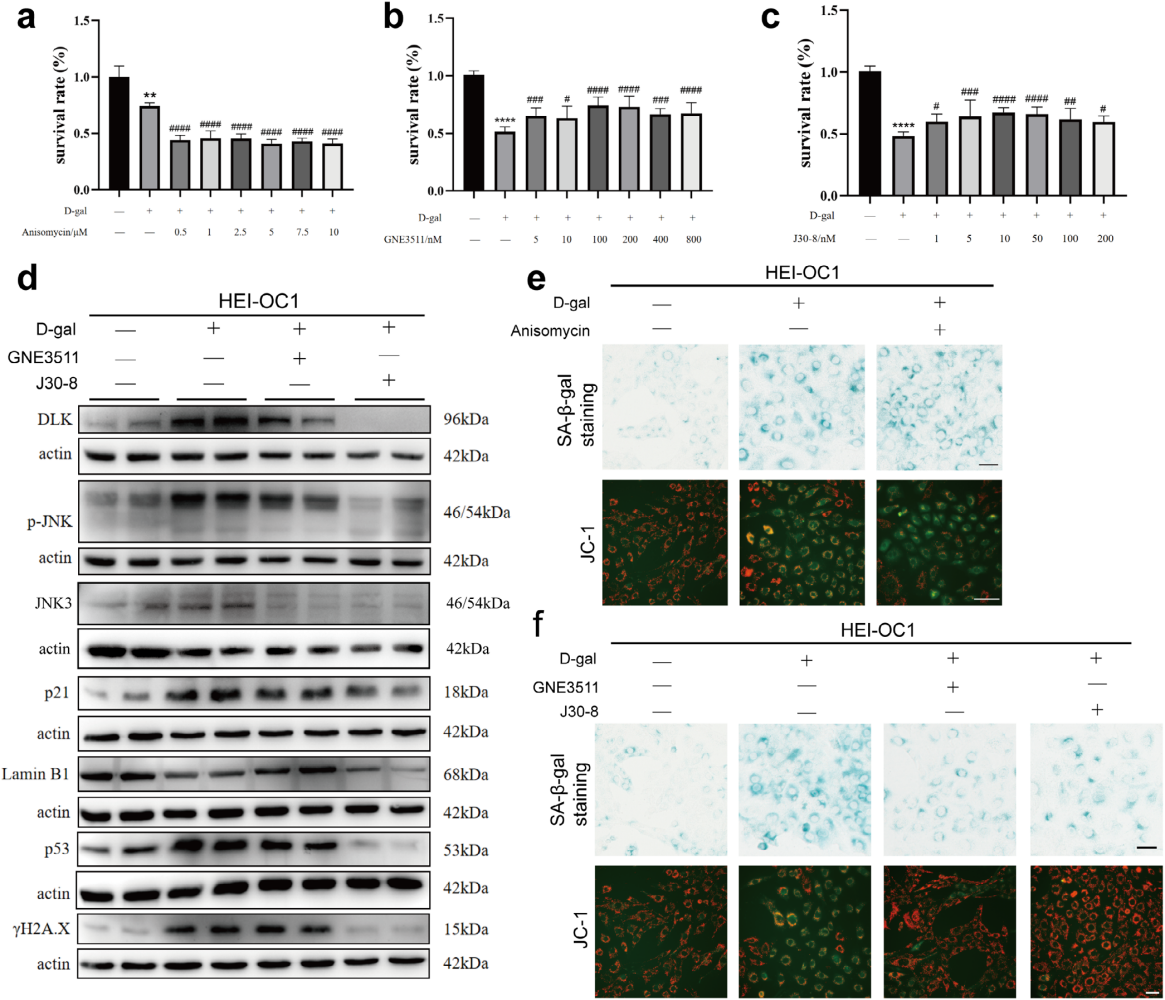
Hair cell senescence can be affected by DLK/JNK3 signal regulation. (a–c) Anisomycin further reduced the survival rate of senescent cells, while DLK inhibitor GNE3511 and JNK3 inhibitor J30‐8 increased the survival rate of senescent cells. (d) After senescent HEI‐OC1 cells were treated with GNE3511 or J30‐8, western blotting showed that the expression of DLK, p‐JNK, JNK3, p21, p53, and γ‐H2A.X decreased and that of LaminB1 increased. (e, f) After treating senescent HEI‐OC1 cells with anisomycin (E), the staining intensity of galactosidase increased, and the mitochondrial membrane potential decreased. Treatment with DLK inhibitor GNE3511 or JNK3 inhibitor J30‐8 (f) produced contrasting results. * indicates comparison with the control group, and # indicates comparison with the d‐galactose‐treated group. Data are presented as mean ± SD, *n* = 3. *p < 0.05, **p < 0.01, ***p < 0.001, ****p < 0.0001. Scale, 50 μm.

After different concentrations of JNK agonist anisomycin were added to d‐galactose‐induced senescent HEI‐OC1 cells, their activity was lower at all concentrations (Figure 6a), and the number of senescence‐related β‐galactosidase positively stained cells increased (Figure 6e, 1st row). JC‐1 detection of the mitochondrial membrane potential showed that anisomycin treatment further decreased the mitochondrial membrane potential of senescent HEI‐OC1 cells, and the red fluorescence disappeared completely in some cells (Figure 6e, 2nd row). While the activity of senescent HEI‐OC1 cells improved after co‐treatment with different concentrations of DLK inhibitor GNE3511 or JNK3 inhibitor J30‐8 (Figure 6b,c), cell senescence was alleviated (Figure 6f).

Western blotting showed that after the induction of HEI‐OC1 cells by d‐galactose, DLK, p‐JNK, and JNK3 expression levels increased. The senescence‐related proteins p21, p53, and γ‐H2A.X increased, and the nuclear cytoskeleton protein LaminB1 decreased, indicating the occurrence of cell senescence. After treatment with the DLK inhibitor GNE3511, the expression of p21 and p53 proteins decreased, and that of LaminB1 increased, implying nuclear skeleton stability. After JNK3 inhibitor J30‐8 treatment, p21, p53, and γ‐H2A.X decreased, indicating that inhibiting the DLK/JNK pathway could ameliorate hair cell senescence (Figure 6d).

### 3.7 Manipulating the DLK/JNK Pathway Can Affect Hair Cell Autophagy Levels

**FIGURE 7:**
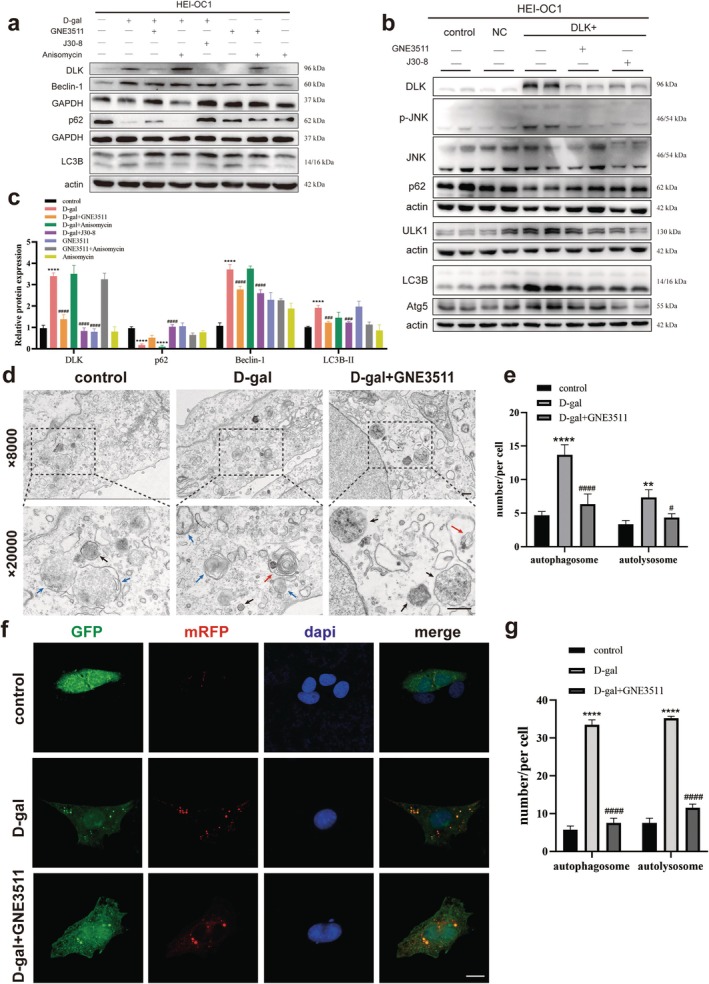
Manipulating the DLK/JNK pathway can affect hair cell autophagy. (a) Western blot showing that autophagy was suppressed after DLK or JNK3 inhibition in senescent HEI‐OC1 cells. (b) Western blot showing that autophagy was suppressed after inhibiting DLK or JNK3 in DLK‐overexpressing HEI‐OC1 cells. (c) The expression of each protein in (a) was quantitatively and statistically analyzed. (d) Lysosomes (black arrow), autophagosomes (blue arrow), and autolysosomes (red arrow) in HEI‐OC1 cells treated with d‐galactose and DLK inhibitor GNE3511 were detected by transmission electron microscopy. (e) Number of autophagosomes and autolysosomes in a single cell in (d). (f) After HEI‐OC1 cells were transfected with mRFP‐GFP‐LC3 lentivirus, they were treated with d‐galactose or DLK inhibitor. Autophagosomes (yellow spots) and autolysosomes (red spots) were observed in each group. (g) Number of autophagosomes and autolysosomes in each group in (f). * indicates comparison with the control group, and # indicates comparison with the d‐galactose‐treated group. Data are shown as mean ± SD, *n* = 3. *p < 0.05, **p < 0.01, ***p < 0.001, ****p < 0.0001. Scale, 5 μm.

In d‐galactose‐induced senescent HEI‐OC1 cells, DLK was significantly upregulated, Beclin‐1 and LC3B‐II were significantly increased, and p62 was significantly decreased. Both GNE3511 and J30‐8 significantly downregulated Beclin‐1 and LC3B‐II expression and upregulated p62 expression (Figure 7a,c). In DLK‐overexpressing HEI‐OC1 cells, the DLK/JNK pathway was activated, ULK1, Atg5, and LC3B‐II expression increased, and p62 expression decreased, suggesting autophagy activation. After GNE3511 and J30‐8 treatment, the DLK/JNK pathway was inhibited, ULK1, ATG5, and LC3B‐II expression decreased, and p62 expression increased, suggesting inhibition of the activated autophagy (Figure 7b).

Transmission electron microscopy showed that autophagosomes and autolysosomes significantly decreased in senescent hair cells due to treatment with the DLK inhibitor GNE3511 (Figure 7d,e). In the d‐galactose‐induced senescent HEI‐OC1 cells, significantly fewer autophagosomes (yellow spots) and autolysosomes (red spots) were observed in a single cell treated with GNE3511 than those in the senescent group (Figure 7f,g). DLK/JNK pathway inhibition has been suggested to inhibit the activation of autophagy in d‐galactose‐induced senescent HEI‐OC1 cells.

### 3.8 DLK/JNK3 Pathway Alleviates Hair Cell Senescence by Inhibiting Autophagy Activation and Activating the Ubiquitin Degradation Process

**FIGURE 8:**
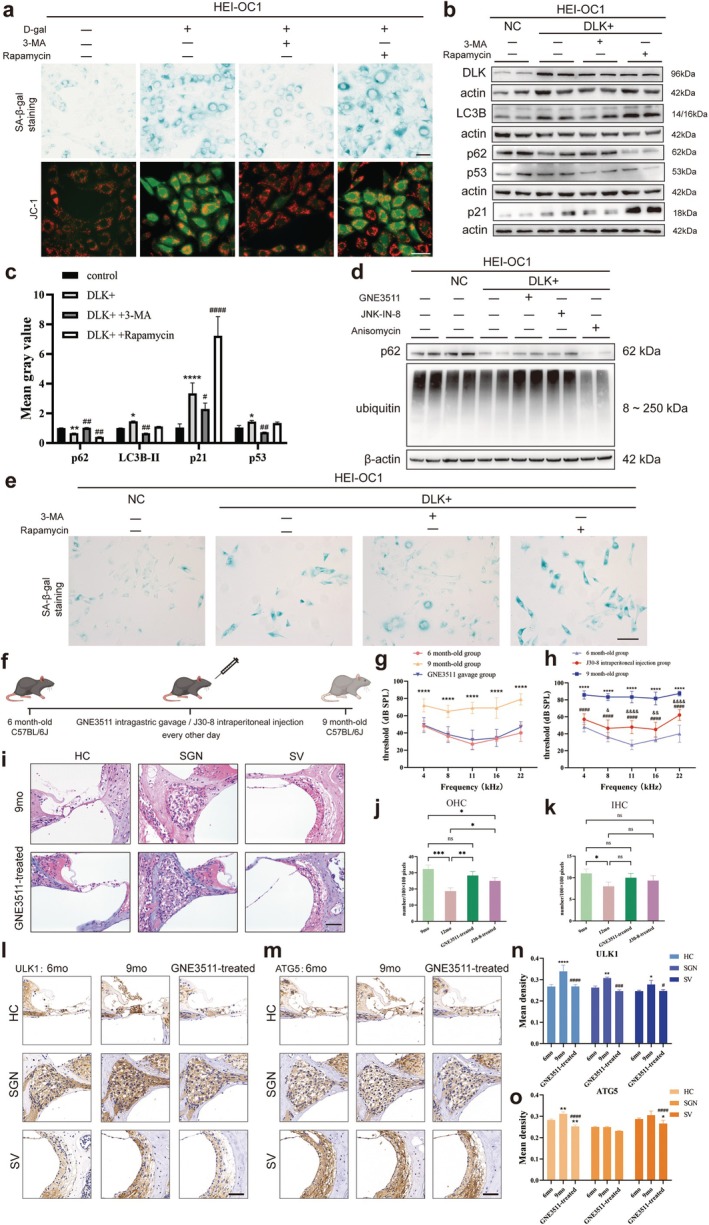
DLK/JNK alleviated hair cell senescence by inhibiting autophagy activation and activating the ubiquitin degradation process, and inhibition of DLK or JNK3 in vivo can alleviate ARHL in mice. (a) In d‐galactose‐induced senescent HEI‐OC1 cells, the positively stained area of galactosidase decreased, and the mitochondrial membrane potential increased after 3‐MA treatment. Contrasting results were observed following rapamycin treatment. (b) Western blot showing that the expression of p53 and p21 decreased after adding 3‐MA to DLK‐overexpressing HEI‐OC1 cells, while that of p21 increased after adding rapamycin. (c) Quantitative and statistical analysis of the expression of each protein in the western blot in (b). (d) Western blot showing that the expression of ubiquitin decreased after DLK overexpression in HEI‐OC1 cells, increased after DLK inhibitor GNE3511 or JNK3 inhibitor J30‐8 treatment, and further decreased after anisomycin treatment. (e) In DLK‐overexpressing HEI‐OC1 cells, the positively stained area of galactosidase staining decreased, and the mitochondrial membrane potential increased after 3‐MA treatment. Contrasting results were observed following rapamycin treatment. (f) Schematic diagram of how DLK inhibitor GNE3511 was administered to C57BL/6J mice. (g) ABR threshold of the GNE3511‐treated group remained the same as that of the 6‐month‐old wild‐type group. (h) ABR threshold of the JNK3 inhibitor J30‐8 group (*n* = 7) was significantly lower than that of the 9‐month‐old wild‐type group (*n* = 5). (i) HE staining showed that the hair cells, spiral ganglion, and stria vascularis in the middle turn of the cochlea in the GNE3511 group were better preserved than those in the 9‐month‐old wild‐type group. (j, k) Number of OHC and IHC in per 100 × 100 pixels area was counted. (l–o) Immunohistochemical staining showing that the expression of ULK1 (l, n) and ATG5 (m, o) in the hair cells, spiral ganglion, and stria vascularis in the GNE3511‐treated group was significantly lower than that in the 9‐month‐old wild‐type group. * indicates comparison with the control group, and # indicates comparison with the DLK‐overexpressing group. Data are presented as mean ± SD, *n* = 3. *p < 0.05, **p < 0.01, ***p < 0.001, ****p < 0.0001. Scale, 50 μm.

When 3‐methyladenine (3‐MA) and rapamycin were added to d‐galactose‐induced senescent HEI‐OC1 cells, the former decreased β‐galactosidase deposition and increased mitochondrial membrane potential. While the latter maintained the state of senescent cells, β‐galactosidase deposition was still significant, and mitochondrial membrane potential was still low (Figure 8a). When 3‐MA was added to DLK‐overexpressing HEI‐OC1 cells, p62 expression significantly increased, whereas that of LC3B‐II, p21, and p53 significantly decreased (Figure 8b,c) and β‐galactosidase deposition decreased (Figure 8e). After rapamycin treatment, p62 significantly decreased, LC3B‐II did not change significantly, p21 significantly increased, p53 did not significantly change (Figure 8b,c), and β‐galactosidase deposition was still significant (Figure 8e). Therefore, inhibiting autophagy can improve the senescent state of hair cells in both d‐galactose‐induced senescent and DLK‐overexpressing HEI‐OC1 cells.

We also examined ubiquitination activity, another pathway for intracellular metabolite degradation. Ubiquitin, a primary marker of ubiquitination, decreased following DLK overexpression and further decreased after JNK activation. However, it increased after inhibition of the DLK/JNK pathway (Figure 8d).

### 3.9 Inhibiting DLK or JNK3 In Vivo Alleviates ARHL Progression in Mice

Six‐month‐old C57BL/6J mice were treated with the DLK inhibitor GNE3511 or JNK3 inhibitor J30‐8 for 3 months (Figure 8f). The ABR results of the three groups showed that the hearing threshold of the GNE3511 group was similar to that of 6‐month‐old mice, and the GNE3511 and 9‐month‐old wild‐type groups significantly differed (Figure 8g). The protective effect of JNK3 inhibition on hearing loss in mice during aging was also evident (Figure 8h). Compared with those of 9‐month‐old wild‐type mice, the hair cell structure and spiral ganglion density of the GNE3511 group were better preserved, considering the middle turn as an example (Figure 8i). We also counted the number of hair cells, and both GNE3511 and J30‐8 exerted significant protective effects on OHC compared to the control group (Figure 8j,k).

As shown in Figure 2, mouse cochlea autophagy levels gradually increased with aging. Following GNE3511 intervention at 6 months of age, the expression of ULK1 and ATG5 in the cochlea of treated mice decreased significantly compared with that in the 9‐month‐old wild‐type group and was maintained at levels akin to those at 6 months of age. Hair cell ATG5 expression was significantly lower than that in the 6‐month‐old wild‐type group (Figure 8l–o).

## 4 Discussion

### 4.1 DLK/JNK3 Pathway Role in Hearing

In the present study, the role of the DLK/JNK3 pathway in ARHL was explored, and the results showed that DLK/JNK3 upregulation aggravated cochlear hair cell senescence and ARHL through excessive autophagy. Based on the results of this study, we hypothesized that DLK is a key regulator of hair cell senescence in mice. Moreover, in vivo, we found that the DLK inhibitor GNE3511 alleviated ARHL in C57BL/6J mice.

The role of the MAPK pathway in different types of hearing loss has also been studied. For example, one of the most important pathways, the JNK pathway, has been studied in hearing loss, especially in noise‐induced hearing loss (Kayyali et al. 2018; Wang et al. 2003). The findings emphasized the negative role of JNK in noise‐induced hearing loss. However, its role in ARHL remains unclear. Three JNK isoforms are known: JNK1, JNK2, and JNK3 (Yang et al. 2012). As we mentioned before, we found in our previous study that the three JNK isoforms were expressed differentially during aging in C57BL/6J mice, and JNK3 was significantly upregulated in aging cochlear hair cells (Ding et al. 2023).

As a member of the MAPKs, JNK3 may be regulated by its upstream MAP3K. Some studies have shown that MAP3K12, also known as DLK, is the upstream regulatory molecule of JNK3 (Niu et al. 2022; Tenenbaum et al. 2021). The DLK/JNK3 pathway plays a key role in mediating neuronal injury (Tenenbaum et al. 2021). DLK/JNK3 pathway inhibition can protect the central and peripheral nervous systems from injury (Patel et al. 2017; Tenenbaum et al. 2021; Watkins et al. 2013). Although DLK is highly expressed in neurons, it has not been explored in auditory neurons or cochlear hair cells. However, few studies have investigated the role of the MAPK pathway in ARHL. For example, the upregulation of p‐p38 and p‐JNK accompanied by oxidative stress has been observed in CBA/J mice during aging (Sha et al. 2009). Furthermore, Benkafadar et al. (2019) observed p38 and p‐p38 upregulation during cochlear cell senescence driven by reactive oxygen species‐induced DNA damage. However, most of these studies have focused on the role of MAPKs in mouse cochlear aging from an oxidative stress perspective (Dong et al. 2024; Guo et al. 2018; Liu et al. 2022) and paid little attention to the relationship between the upstream MAPK pathway and the occurrence and progression of cochlear senescence. Our study showed that the DLK/JNK3 pathway is significantly upregulated during mouse cochlear senescence, possibly accelerating hair cell senescence. However, further research is needed to determine the specific mechanisms by which DLK activates JNK3, such as through phosphorylation or other modifications. Additionally, our study observed that inhibition of the DLK/JNK3 pathway also exerted a protective effect on SGN. This suggests that the auditory system protection mediated by DLK/JNK3 suppression may not be exclusively dependent on hair cell mechanisms. The mechanism underlying the pathway's protection of SGN requires further investigation and may differ from the autophagy‐mediated protection observed in hair cells.

### 4.2 Abnormal Activation of Hair Cell Autophagy Mediated by DLK Leads to Senescence

Many studies have shown that the MAPK pathway plays a key role in mediating cell death caused by autophagy (Sui et al. 2014). Borsello et al. (2003) found that acute toxic neuronal death in the mouse hippocampus is caused by abnormal autophagy activation mediated by the MAPK pathway. Zhang et al. (2019) found that the anti‐cancer drug β‐thujaplicin mediated abnormal activation of liver cancer cell autophagy and led to cell death through reactive oxygen species activation of the p38MAPK pathway. Li et al. (2009) reported that activation of the MAPK pathway in the human nasopharyngeal carcinoma cell line CNE‐2 and the hepatocellular carcinoma cell line Hep3B could mediate the expression of Beclin1, an important autophagy protein that can abnormally activate autophagy, thus inducing the autophagic death of tumor cells.

In audiology, especially in ARHL, little research has been conducted on the MAPK pathway regulation of autophagy. Owing to the dual role of autophagy (Baehrecke 2005; Kocaturk et al. 2019; Li et al. 2015), its role in physiological and pathological processes may depend on its degree of activation. Wang et al. (2021) found that pretreatment with the ERK1/2 inhibitor U0126 significantly reduced cisplatin‐induced hair cell apoptosis and autophagy in HEI‐OC1 cells and the organ of Corti and also decreased cisplatin‐induced reactive oxygen species production and mitochondrial membrane potential. However, Yang et al. (2018) found that in cisplatin‐induced ototoxic hearing loss, both JNK inhibition and autophagy activation protected hair cells and spiral ganglia from cisplatin ototoxicity. Generally, the role of autophagy homeostasis regulated by the MAPK pathway in hearing loss, especially ARHL, requires further investigation. The present study focused on the role of DLK‐mediated autophagic dysfunction in ARHL.

DLK is an important biomarker and potential target for some neurodegenerative diseases (Le et al. 2023; Maes et al. 2023; Wlaschin et al. 2023). In a mouse model of spinal dorsal root ganglion injury, DLK was shown to be a core regulatory factor that mediates a series of injury responses, including axonal regeneration and neuronal apoptosis, which are necessary for activating regenerative transcription in response to peripheral nerve injury (Shin et al. 2019). Huntwork‐Rodriguez et al. (2013) reported that in addition to DLK activating JNK, the positive feedback of JNK on DLK phosphorylation also occurs during spinal cord axonal injury. Furthermore, decreased levels of DLK ubiquitination were observed during the removal of nutritional factors, suggesting that DLK signaling may be continuously activated in this process. Therefore, DLK can be regarded as an effector that triggers downstream cellular responses (primarily JNK/c‐Jun) in various neurodegenerative diseases.

However, the possible role of DLK in auditory cell degeneration has not yet been reported. Therefore, in the current study, we explored this area and found that DLK is expressed in the cochlea, especially in hair cells, and DLK/JNK‐mediated activation of autophagy may aggravate senescence in hair cells. To investigate the mechanism further, future research should aim to identify the specific regulator within the autophagy process that is directly regulated by the DLK/JNK3 pathway. Future studies can employ inner ear‐specific conditional knockout mouse models targeting this regulatory molecule for further in vivo validation (Xu et al. 2023). In addition to naturally aged mouse models, similar DLK/JNK3 pathway alterations can be further validated in d‐galactose‐induced aging mouse models and rat models (Li et al. 2018; Wu et al. 2012, 2014; Zhong et al. 2012).

### 4.3 Role of Autophagy and Ubiquitination in ARHL

Autophagy and the ubiquitin‐proteasome system (UPS) are the two main mechanisms by which cells remove intracellular waste (Varshavsky 2017). They coordinate at multiple levels to ensure cellular health and appropriate responses to stress and injury (Pohl and Dikic 2019). Macrophage autophagy (macroautophagy) is the main type of autophagy that has been the focus of many studies. Autophagy is a highly conservative cellular mechanism (Parzych and Klionsky 2014). After external stress, cells activate the ‘autophagy’ program through signal transduction and regulation (Mizushima and Komatsu 2011). Thus, ‘autophagy’ removes harmful substances and damaged organelles in the cell to maintain normal physiological function (Mizushima and Komatsu 2011). Excessive autophagy activation mediates cell senescence, degeneration, and finally death (Schwartz 2021), as well as tissue damage and dysfunction. Therefore, the balance of autophagy is important for maintaining self‐renewal and a stable internal cellular environment.

Many studies have confirmed the important role of autophagy in neurodegenerative diseases (Cai and Ganesan 2022; Fleming et al. 2022; Wang and Xu 2020). In audiology, studies on autophagy and hearing loss have been conducted, but they have mostly focused on ototoxic and noise‐induced hearing loss (Song et al. 2024; Yuan et al. 2015). In these studies, moderately enhanced autophagy often appeared to favor cellular metabolic waste removal and cell survival (He et al. 2021, 2023). However, some studies have reported the harmful effects of overactivated autophagy in cells. For instance, Jian et al. (2021) found that ferroptosis occurred in cisplatin‐treated HEI‐OC1 cells and was dependent on autophagy activation. Blocking autophagy significantly alleviated cisplatin‐induced cell death. Tang, Sun, et al. (2021) demonstrated that caffeine induces autophagy and apoptosis in HEI‐OC1 hair cells through the SGK1/HIF‐1α pathway, resulting in hearing loss in mice. In summary, researchers have observed different roles of autophagy in hair cell death in different injury models. Autophagy disorder, a characteristic of cell senescence (Herranz and Gil 2018), may also be one of the initial factors mediating cell senescence. Therefore, in the present study, we focused on autophagy. Upregulation of DLK can activate autophagy, and autophagy is suppressed by inhibiting the DLK/JNK pathway. Based on our previous research, we focused on JNK3 in the JNK pathway and speculated that excessive autophagy activation induced by JNK3 and mediated by DLK leads to hair cell senescence. Barutcu et al. (2018) showed that JNK1/2 did not play a significant role in hunger‐induced autophagy in mouse embryonic fibroblasts, further confirming that the autophagy observed was probably mainly mediated by JNK3 rather than JNK1/2. However, this must be verified in future studies.

In the present study, autophagy increased with senescence. To clarify whether this increase was a protective factor against senescence or an initiating factor causing aging, we regulated autophagy and observed that the level of cell senescence was reduced after inhibiting autophagy. Therefore, excessive activation of autophagy promoted cell senescence in our model. We assumed that at the beginning of hair cell senescence, autophagy played a protective role as usual. However, sustained activation of autophagy was detected in our model (Figure S2) and could probably be harmful to hair cells. As a mechanism for eliminating intracellular metabolites and harmful substances, functional compensation for autophagy occurs when autophagy is inhibited, thereby reducing cell senescence.

The UPS is another intracellular degradation program whose substrate ubiquitination shares a degradation signal similar to that of autophagy (Kwon and Ciechanover 2017). Competition between the two mechanisms is determined by the affinity of the complex rather than by the individual affinity of the receptor for ubiquitin (Lu et al. 2017). Notwithstanding, these two pathways interact with each other. For example, the proteasome itself is recognized as a substrate for autophagy and degradation, where the p62 domain bound to the proteasome plays a decisive role in whether the proteasome is degraded (Cohen‐Kaplan et al. 2016). Increasing evidence suggests that autophagy and UPS are simultaneously affected by disease and interact with each other (Popovic et al. 2014). In the present study, changes in ubiquitination levels were observed during hair cell senescence and were in a complementary balance with autophagy levels under stress. Therefore, we speculate that p62 and ubiquitin, as bands connecting the UPS and autophagy, respond through complex intracellular regulatory mechanisms. UPS activation compensates for autophagy inhibition. At present, the balance between autophagy and the UPS has not been elucidated, and the results of this study can be used as a reference.

## Author Contributions

**Rui Ding:** data curation, writing – original draft. **Weiyi Huang:** formal analysis, validation. **Chenling Shen:** software, conceptualization. **Yi Pan:** methodology. **Yiming Zhong:** validation. **Bing Kong:** validation. **Yilin Shen:** supervision. **Mingliang Xiang:** project administration, writing – review and editing, funding acquisition. **Bin Ye:** writing – review and editing, resources, funding acquisition.

## Disclosure

The authors have nothing to report.

## Conflicts of Interest

The authors declare no conflicts of interest.

## Supporting information


**Figure S1.** Cellular immunofluorescence showing that autophagy was excessively.
**Figure S2.** HEI‐OC1 cells treated with increasing concentrations of D‐galactose.

## Data Availability

The data that support the findings of this study are available on request from the corresponding author.
