# Supplementary material for: DLK/JNK3 Upregulation Aggravates Hair Cell Senescence in Mice Cochleae via Excessive Autophagy
Source: Aging Cell. 2025 May 12;24(8):e70099. doi: 10.1111/acel.70099 (PMC12341777; doi:10.1111/acel.70099)
Supplement: Supplementary file 1 — Figure S1. Cellular immunofluorescence showing that autophagy was excessively. Figure S2. HEI‐OC1 cells treated with increasing concentrations of D‐galactose. [file ACEL-24-e70099-s001.pdf]

Supplement

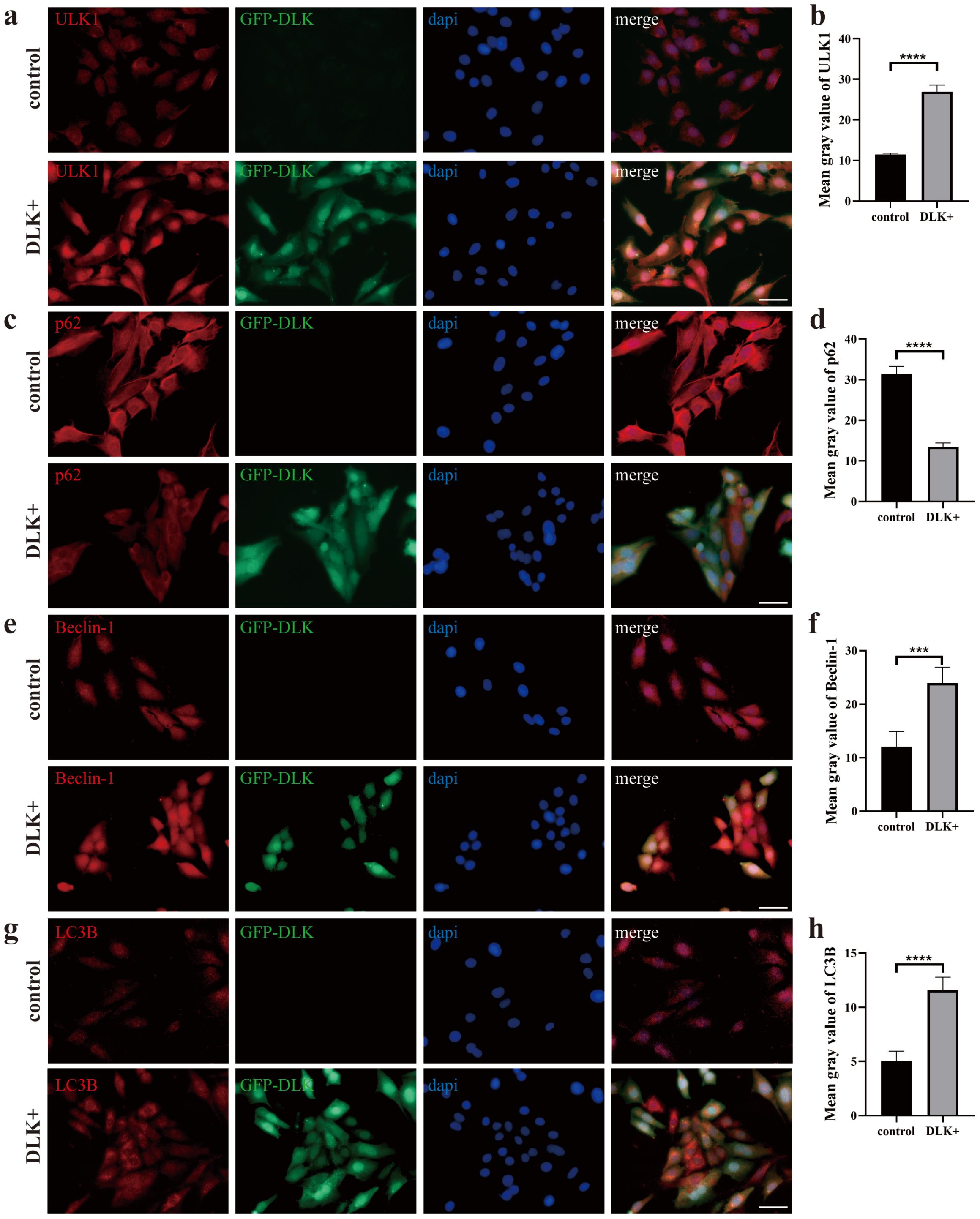

Figure S1. Cellular immunofluorescence showing that autophagy was excessively activated in DLK-overexpressing HEI-OC1 cells. In the control and DLK-overexpressing groups, the expression of ULK1 (**a** and **b**), p62 (**c** and **d**), Beclin-

1 (**e** and **f**), and LC3B (**g** and **h**) proteins was determined by immunofluorescence, and quantitative statistics showed that ULK1, Beclin-1, and LC3B increased, whereas p62 decreased. Data are presented as mean ± SD, $n = 3$. ***$P < 0.001$, ****$P < 0.0001$. Scale: 50 μm.

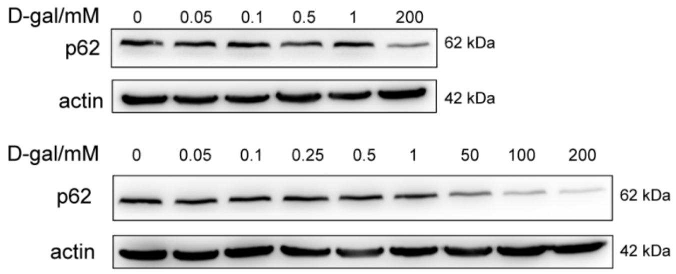

Figure S2. HEI-OC1 cells treated with increasing concentrations of D-galactose showed decreasing expression of p62 protein, indicating increasing level of autophagy.
